# 

**Published:** 1881-11

**Authors:** 


### CONTENTS.

*ORIGINAL:*

PAGE.

The British Medical Association and Homœopathy, . . . . 509

The Scientific Use of the Nosodes. E. W. Berridge, M. D., London, . 516

Isopathy: A Fatal Error. Ad. Lippe, M. D., Philadelphia, . . . 528

Treatment of President Garfield. T. Dwight Stow, M. D., Fall River, Mass., . . . . . . . . . . . . 533

A New Discovery in the Service of Homœopathy. Translated from "*Populare Zeitschrift fur Homœopathy,*" Jan. 1881, by Prof. Manuel Græter, . . . . . . . . . . . . 538

Diphtheria, Bacteria, and Dr. Gregg. P. P. Wells, M. D., Brooklyn, N. Y., . . . . . . . . . . . . 546

A False Accusation, . . . . . . . . . . . 550

*CLINICAL BUREAU:*

High Potencies in Parturition. Julius Schmitt, M. D., Rochester, N. Y., 551
A Curious Case.—Cantharis. William A. Hawley, M. D., Syracuse, N. Y., . . . . . . . . . . . . 554
PERISCOPE, . . . . . . . . . . . . 557

*BOOK NOTICES, REVIEWS, ETC.*

Homœopathic Therapeutics, as applied to Obstetrics. Sheldon Leavitt, M. D., . . . . . . . . . . . . 559
The Guiding Symptoms of our Materia Medica. Vol. III. C. Hering, 559

